# Application of Inelastic Neutron Scattering to the Methanol-to-Gasoline Reaction Over a ZSM-5 Catalyst

**DOI:** 10.1007/s10562-016-1742-5

**Published:** 2016-04-15

**Authors:** Russell F. Howe, James McGregor, Stewart F. Parker, Paul Collier, David Lennon

**Affiliations:** 1 Department of Chemistry, University of Aberdeen, Aberdeen, AB24 3UE UK; 2 Department of Chemical and Biological Engineering, University of Sheffield, Sheffield, S1 3JD UK; 3 ISIS Facility, STFC Rutherford Appleton Laboratory, Chilton, Oxon OX11 0QX UK; 4 Johnson Matthey Technology Centre, Blounts Court, Sonning Common, Reading, RG4 9NH UK; 5 School of Chemistry, University of Glasgow, Joseph Black Building, Glasgow, G12 8QQ UK

**Keywords:** ZSM-5, Methanol, Inelastic neutron scattering, Hydrocarbon pool

## Abstract

**Abstract:**

Inelastic neutron scattering (INS) is used to investigate a ZSM-5 catalyst that has been exposed to methanol vapour at elevated temperature. In-line mass spectrometric analysis of the catalyst exit stream confirms methanol-to-gasoline chemistry, whilst ex situ INS measurements detect hydrocarbon species formed in/on the catalyst during methanol conversion. These preliminary studies demonstrate the capability of INS to complement infrared spectroscopic characterisation of the hydrocarbon pool present in/on ZSM-5 during the MTG reaction.

**Graphical Abstract:**

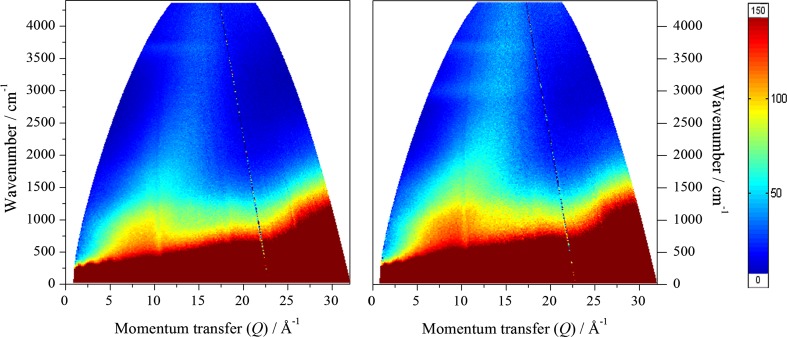

## Introduction

The conversion of alcohols to hydrocarbons was first introduced in the Mobil methanol-to-gasoline (MTG) process using an HZSM-5 catalyst, commercialised in New Zealand in 1986. Lurgi’s methanol to olefins (MTO) process, also using HZSM-5, UOP-Statoil’s MTO process using a SAPO-34 catalyst, and Topsoe’s improved gasoline synthesis (TIGAS) using a proprietary zeolite catalyst followed. The availability of cheap methanol derived from natural gas was the initial driver for these technologies. More recently, methanol derived from coal has become the source of transport fuels or olefin feedstocks via MTG or MTO type processes. In the future biomass and other renewable resources are likely to provide a ready supply of methanol, e.g. through gasification and subsequent hydrogenation of CO/CO_2_, or via direct oxidation of methane produced by aerobic digestion. MTG and MTO processes therefore provide a route to fuels and chemicals from renewable feedstocks.

The past 30 years have seen numerous investigations of the reaction pathways and mechanisms by which methanol is converted to hydrocarbons over acid zeotype catalysts, as reviewed recently for example in references [1–4]. Three different components of the reaction pathway can be distinguished: (1) the initial reaction steps in which methanol reacts with acid sites in the zeolite or SAPO catalysts; (2) the formation of hydrocarbon products during steady-state working conditions; and (3) the catalyst deactivation through so-called coke formation. As in any catalytic system, understanding the reaction pathway is essential if optimum product selectivity and catalyst performance are to be achieved.

Most attention in the last 10 years has focussed on the reactions occurring under steady-state working conditions. The so-called ‘hydrocarbon pool’ mechanism has found widespread support [2, 5–7]. In this mechanism, two catalytic cycles operate in parallel: alkenes are methylated and subsequently cracked in one cycle, while aromatics are methylated and subsequently dealkylated in the other. Experimental support for this mechanism has come from, for example, ^13^C labelling studies, NMR and UV–VIS identification of polymethyl aromatic species occluded in working catalysts, and post reaction analysis of occluded species liberated from used catalysts by GC–MS. The differences in product distribution found between zeolites with different pore sizes have been rationalised in terms of different contributions from the two different cycles.

The mechanisms by which the hydrocarbon pool is initially formed from methanol are much less clear-cut (and this subject has been comprehensively reviewed in [1]). Infrared spectroscopy has been extensively used to investigate species formed when methanol first contacts the zeolite catalyst, and clear evidence obtained for the formation of reactive methoxy groups from reaction of methanol or dimethylether with Brønsted acid sites [8–12]. After longer reaction times at higher temperatures more complex infrared spectra develop which have been assigned variously to adsorbed methylaromatics and olefinic species [12, 13]. Infrared spectroscopy on ZSM-5 is limited to the energy range 1350–4000 cm^−1^ as a consequence of the intense absorption bands below 1350 cm^−1^ due to Si–O and Al–O stretching vibrations of the zeolite framework (although Qian et al. [12] were able to observe a small window in the spectrum of SAPO-34 between 800 and 900 cm^−1^). A second limitation of the FTIR technique is that catalysts at a later stage in the reaction path become difficult to observe because of the presence of strongly absorbing species [13]; i.e. the catalysts become dark in colour. One alternative means of accessing the full vibrational spectrum of the catalyst at all stages of the reaction coordinate is to employ the technique of inelastic neutron scattering (INS) [14].

The use of INS to obtain vibrational spectra of molecular systems has been reviewed by Parker et al. [15, 16]; highlighting how direct geometry INS spectrometers, e.g. MAPS [17], can provide supplementary and additional information to that achievable with indirect geometry INS spectrometers, e.g. TOSCA [17]; the latter being the more typical instrument of choice for molecular spectroscopy investigations by INS. That work also outlines an increasing trend of using of INS to examine a variety of heterogeneously catalysed reaction systems [15]. For example, following on from the work of Silverwood et al. who used INS to examine alumina-supported nickel catalysts applied to the dry [18–21] and steam [22] reforming of methane, Warringham and co-workers have used a combination of indirect [23] and direct [24–26] geometry INS spectrometers to investigate iron based Fischer–Tropsch synthesis (FTS) catalysts. Moreover, Warringham et al. [27] recently reported on sample environment details relevant to the acquisition of INS spectra of heterogeneous catalysts.

Against this background, it is timely to consider whether INS can be applied to look at MTG and/or MTO chemistry over zeolitic materials such as ZSM-5. Given the ability of INS to selectively probe hydrogeneous vibrational modes [15], it is enticing to discover whether the technique can provide new information on the adsorbed hydrocarbon species present at various stages of the methanol to hydrocarbon reaction. Very recently, O’Malley et al. [28] have used a combination of neutron scattering methods matched by ab initio calculations to investigate, amongst other things, the reaction of methanol with ZSM-5 at room temperature. That study showed methanol to be immobilized due to methoxylation, and that the formation of adsorbed methoxy groups is facile over this material at room temperature. In this paper, we present a preliminary report on the INS spectra of a commercial grade ZSM-5 catalyst exposed to a methanol feedstream at 623 K. With mass spectrometry indicating representative MTG chemistry, the reaction was stopped, quenched by rapid cooling to room temperature in flowing helium, and the catalyst sample taken to the INS spectrometer without exposure to air for spectral acquisition. The resulting ex situ spectra unambiguously reveal the presence of hydrocarbon species, which may be associated with a hydrocarbon pool. Whereas O’Malley et al. [28] concentrated on methanol exposure to ZSM-5 at room temperature, this communication describes an investigation performed at reaction temperatures representative of those utilised in MTG unit operations. Thus, INS is being used here to assess how hydrogen is partitioned within the catalyst matrix during conditions that correspond to the commercially relevant active phase of MTG operation. Although further work is necessary to examine spectra at different reaction times and to better correlate catalytic performance with the vibrational spectra, this short communication demonstrates the capability of INS to positively contribute to the understanding of this economically relevant but technically challenging reaction system.

## Experimental

A commercial grade ZSM-5 catalyst (ACS Material LLC, USA; SiO_2_/Al_2_O_3_ molar ratio, 38:1, specific surface area ≥250 m^2^ g^−1^, pore volume ≥0.25 ml g^−1^, pore size ~5 Å, bulk density ~0.72 kg l^−1^) supplied in the form of column shaped pellets (diameter 2 mm, length 2-10 mm) was employed. Two Inconel reactors [29] were charged with the catalyst pellets. The first reactor was attached to a gas manifold apparatus [27] and the catalyst dried by the following procedure: whilst continually maintaining a flow of helium over the catalyst (1000 sccm, CK Gas, >99.0 %), the sample was heated to 623 K at a heating rate of 10 K min^−1^ and then maintained at that temperature for 2 h. The heating was stopped and the sample allowed to cool to ambient temperature in a continuous flow of He. The reactor was then isolated and transferred to an argon-filled glove box (MBraun UniLab MB-20-G, [H_2_O] < 1 ppm, [O_2_] < 2 ppm) for loading into an aluminium INS cell that is sealed via an indium wire gasket [17]. The second sample was similarly dried but the dehydration stage was followed by dosing of the catalyst with methanol vapour by means of a bubbler arrangement whilst the catalyst was maintained at 623 K. The eluting stream was analysed by in-line mass spectrometry (Hiden Analytical, HPR-20) with the spectrometer connected to the reactor exit line via a differentially-pumped, heated quartz capillary. The apparatus is equipped with a ‘catch-pot’ downstream of the catalyst. Material collected there was subsequently analysed by GC–MS. After approximately 6 h on-stream, the methanol flow and heating were stopped, and the sample allowed to cool to ambient temperature in a continuous flow of He. The reactor was then isolated and transferred to an aluminium INS cell using the procedure outlined above. The two samples (dried catalyst and reacted catalyst) were then transported to the INS spectrometer and the spectrum recorded at 20 K.

A series of spectra were originally measured on the MAPS spectrometer using the high resolution A chopper package operating at incident energies of 4840 (frequency = 600 Hz) and 2016 cm^−1^ (frequency = 400 Hz) respectively. However, the spectrum of the reacted sample exhibited a poor signal:noise ratio, indicating a low degree of hydrogen retention by the catalyst. A previous use of INS by Warringham and co-workers to examine the hydrogenation of propyne over alumina-supported Pd catalysts showed how the enhanced sensitivity of the MERLIN spectrometer could be used to acquire the vibrational spectra of minority species, albeit at reduced resolution compared to that attainable with the MAPS spectrometer [30]. Consequently, in an attempt to obtain superior spectra, both the dried and reacted samples were also run on the MERLIN spectrometer using the low resolution S chopper package operating at incident energies of 4840 (frequency = 600 Hz) and 2016 cm^−1^ (frequency = 400 Hz) respectively. Results are presented from both spectrometers.

## Results and Discussion

### Reaction Testing

**Fig. 1:**
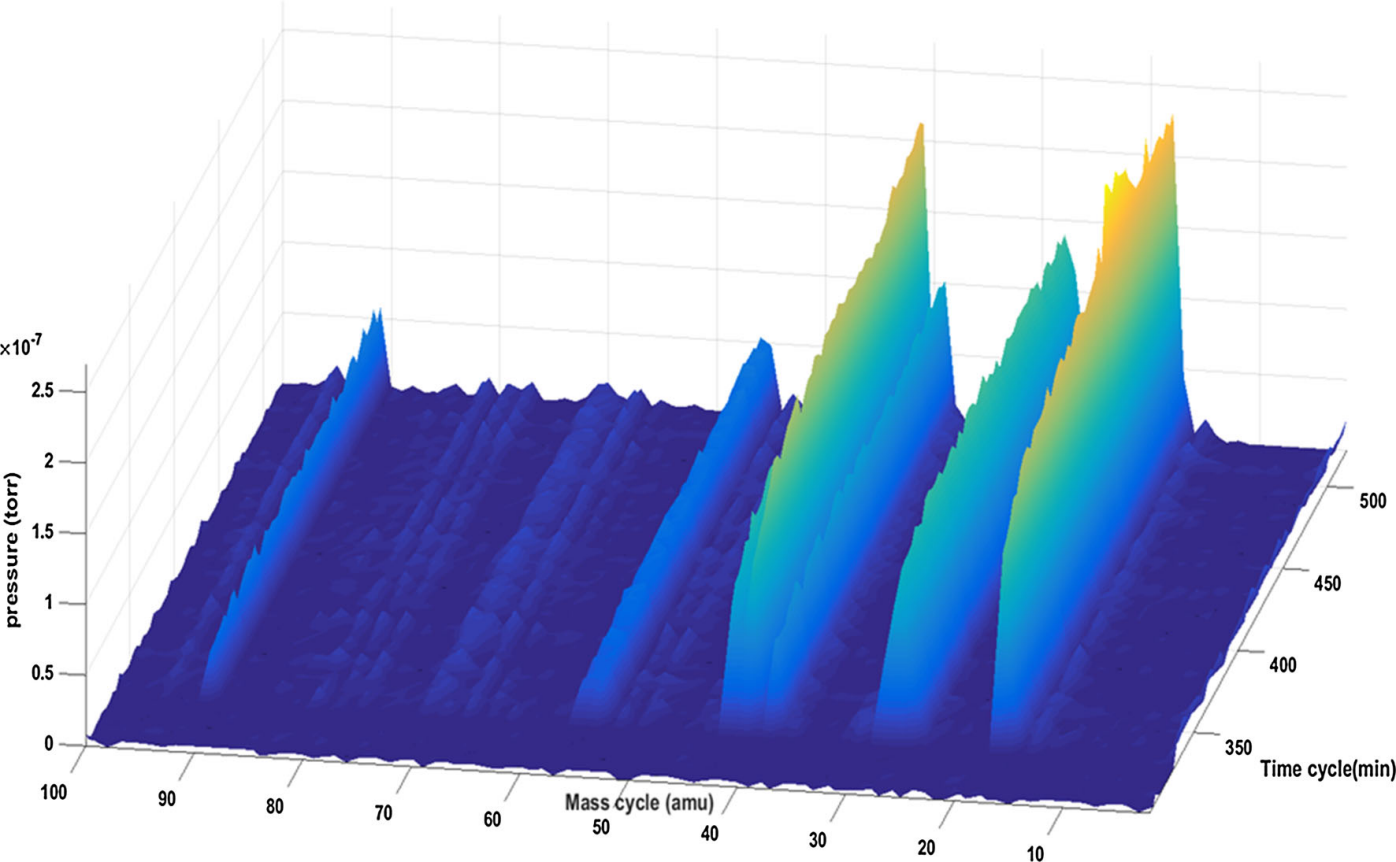
Mass spectrometer profile for the catalyst exit stream following the induction period

Mass spectrometry showed the reaction system to exhibit an induction period lasting *ca*. 3 h, followed by operation at approximately steady-state conditions for the remainder of the reaction period investigated (3 h). Figure 1 shows the mass spectrometer profile for the catalyst exit stream after the induction period. The major peaks identified have m/z ratios of: 18 (H_2_O); and 28 (C_2_H_4_,); 41 (C_3_H_5_); 55 (C_4_H_7_, C_3_H_3_O); and 91 (C_7_H_7_). These features indicate the formation of longer-chained hydrocarbons and oxygenates; while the m/z = 91 peak is indicative of methylated benzenes, e.g. toluene, xylenes, etc. the complementary GC–MS analysis of the catch-pot liquid, collected post-reaction, shows some unconverted methanol, with smaller amounts of ethanol, alongside species identified as methylated benzenes (ranging from toluene through to hexamethylbenzene). In addition, a small quantity of heavier products, that may be substituted naphthalenes, were also present.

Collectively, these analytical results indicate that after 6 h on-stream the catalyst is operating in a pseudo-steady state regime, whereby methanol is converted to a range of higher molecular weight products that includes C_2_ and C_3_ olefins, as well as a number of alkylated benzenes. This distribution is typical of previous reports of MTG chemistry over ZSM-5 [1–3], although it is recognised that the catalyst is in the early stages of its reaction cycle with, for example, little evidence of a coking stage active over the short reaction period studied. This assumption is consistent with the observation that, post-reaction, the originally white pellets had changed to a mild grey colour; cutting across the pellet column revealed that the grey colouration was only present at the catalyst surface, with the inside of the pellets retaining their original clean white colouration. The presence of retained carbonaceous material on or within the catalyst was confirmed by temperature programmed oxidation (TPO). This indicated the presence of a relatively small quantity of carbonaceous species, however the oxidation temperature ranged from 473 to 973 K, thereby indicating that at least some of the species showed significant complexity and order; beyond that expected for short linear hydrocarbons. TPO results are therefore consistent with the presence of aromatic species in the post-reaction sample.

### Inelastic Neutron Scattering

INS is inherently a two-dimensional spectroscopy, being able to sample both energy (E, cm^−1^) and momentum transfer (Q, Å^−1^) [15, 16]. This attribute is particularly accessible on direct geometry instruments such as MAPS and MERLIN [15, 16]. A commonly adopted means of visualising this information is to use “mitre” plots [26], where the neutron scattering intensity is plotted as a function of energy (ω, y axis) and momentum transfer (Q, x axis).

**Fig. 2:**
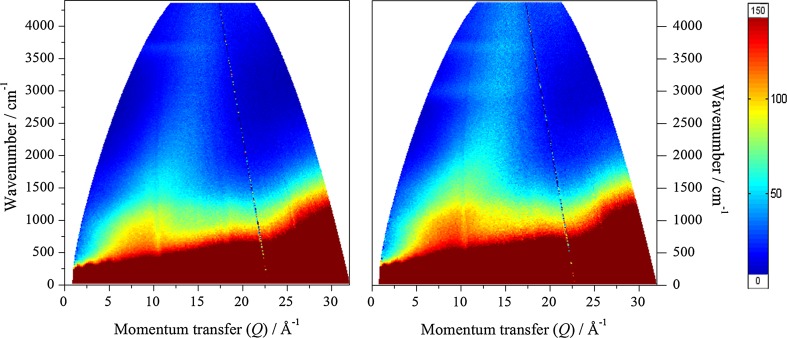
*S*(*Q*,*ω*) plots recorded on the MERLIN spectrometer for the clean (*left*) and reacted (*right*) samples with 4840 cm^−1^ incident energy

**Fig. 3:**
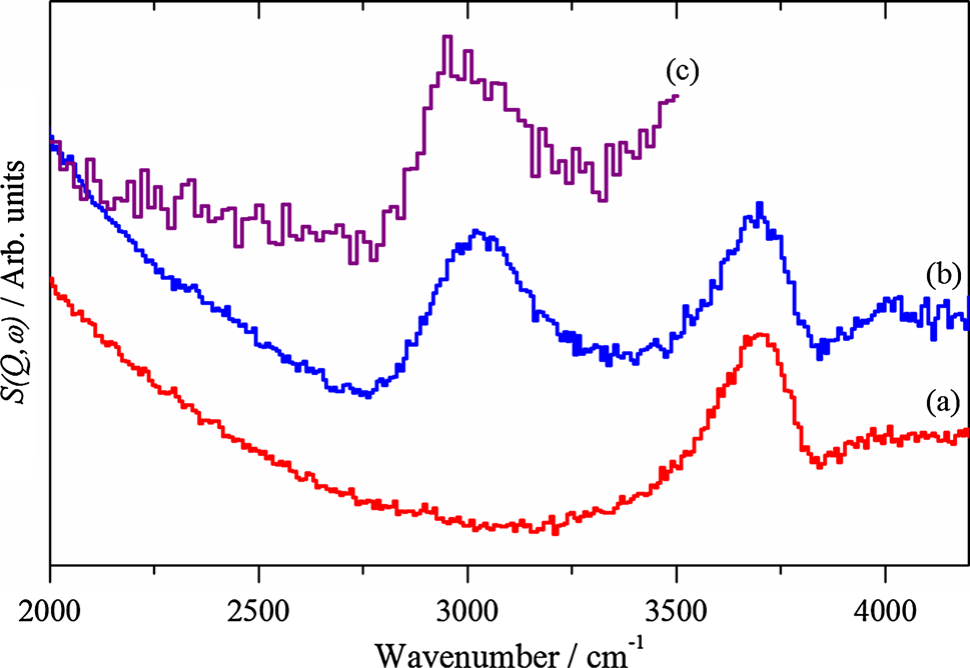
INS spectra of the (*a*) clean and (*b*) reacted samples recorded with 4840 cm^−1^ incident energy using the MERLIN spectrometer. Spectrum (*c*) is the reacted sample recorded using the MAPS spectrometer recorded with 4840 cm^−1^ incident energy

**Fig. 4:**
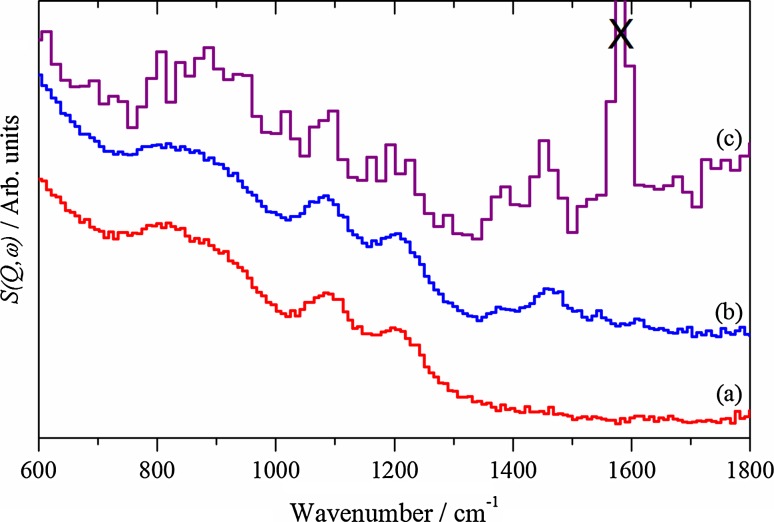
INS spectra of the (*a*) clean and (*b*) reacted samples recorded with 2016 cm^−1^ incident energy using the MERLIN spectrometer. Spectrum (*c*) is the reacted sample recorded using the MAPS spectrometer recorded with 2016 cm^−1^ incident energy

Figure 2 shows the *S*(*Q*,*ω*) mitre plots recorded on the MERLIN spectrometer for the clean (left hand frame) and reacted (right hand frame) samples with 4840 cm^−1^ incident energy. The reacted sample exhibits more intensity than the dried catalyst, indicating retention of hydrogenous species on reaction, consistent with the results of TPO. Further, there is a band of intensity at ~3000 cm^−1^, in addition to a feature at ~3600 cm^−1^ that is present in both samples. Figures 3 and 4 show the spectra integrated over the low Q range 0 ≤ *Q* ≤ 12 Å^−1^; this results in the more conventional presentation of INS spectra (a two-dimensional plot of neutron scattering intensity, S(ω), versus energy).

Initial inspection of Figs. 3 and 4 shows the superior sensitivity of the MERLIN acquired spectra (a and b) compared to the MAPS spectrum (c). The MERLIN spectrum of the clean zeolite above 2000 cm^−1^ (Fig. 3a) shows a single band centred just below 3700 cm^−1^. This is due to O–H stretching vibrations of the zeolite. Infrared spectra of dehydrated HZSM-5 show up to 3 component bands in this region, at around 3740 cm^−1^ (SiOH associated with external silanol groups), 3660 cm^−1^ (AlOH associated with extra-framework aluminium species) and 3610 cm^−1^ (the Brønsted acid, Si(OH)Al) [9]. The INS experiment lacks the spectroscopic resolution to resolve these species. The MERLIN spectrum of the clean zeolite in the region 1800-600 cm^−1^ (Fig. 4a) shows three bands at ~800, 1080 and 1200 cm^−1^. This is closely similar to the spectrum of dehydrated HZSM-5 reported in this region by Jobic et al. [31]. These authors assigned a band at 1080 cm^−1^ to the in-plane deformation of bridging OH groups (the Brønsted acid sites). A shoulder at 1210 cm^−1^ was attributed to either a combination of the in-plane OH deformation with a low frequency lattice mode, or possibly an in-plane deformation of a second type of hydroxyl group, while a band at 785 cm^−1^ was assigned to a framework mode coupled with proton motion. In dehydrated HNaY, on the other hand, a shoulder at ~860 cm^−1^ has been assigned to the in plane deformation mode of silanol groups [32].

The MERLIN spectrum of the zeolite catalyst following reaction in methanol shows one additional strong band in the region 2000–4000 cm^−1^ centred at 3000 cm^−1^ (Fig. 3b). This is due to C-H stretching vibrations of adsorbed hydrocarbon species, but the spectral resolution of MERLIN does not allow a differentiation between aliphatic (<3000 cm^−1^) and olefinic or aromatic (>3000 cm^−1^) CH groups. The higher resolution of the MAPS spectrum (Fig. 3c) suggests that both are present (a peak at 2960 cm^−1^ with a higher frequency shoulder at ~3080 cm^−1^). In the 600–1800 cm^−1^ region both the MERLIN (Fig. 4b) and MAPS (Fig. 4c) spectra show new bands at 1380 and 1450 cm^−1^.

Together, Figs. 3 and 4 indicate the presence of aliphatic species in/on the catalyst. Bands at 1380 and 1460 cm^−1^ are typical of the symmetric and antisymmetric deformations of methyl groups. In their infrared study of ZSM-5 exposed to methanol at 370 °C Palumbo et al. [13] attributed bands at 1380 and 1465 cm^−1^ to methyl groups in methyl substituted benzene rings, along with bands between 1500 and 1700 cm^−1^ due to C=C stretching vibrations of such species. No aromatic C–H stretching modes above 3000 cm^−1^ were detected in the infrared study of Palumbo et al., which was attributed to the complete methyl substitution of the aromatic rings. The shoulder detected above 3000 cm^−1^ in the MAPS spectrum (Fig. 3c) indicates however that some sp^2^ hybridised C–H bonds, olefinic or aromatic, are present. In INS the intensity is directly proportional to the number of C-H oscillators, not their type [15], whereas in infrared spectroscopy the extinction coefficients can be widely different. Thus, INS is revealing the presence of hydrocarbonaceous species not readily observable by optical spectroscopy.

These preliminary experiments have demonstrated that INS can directly observe hydrocarbon species present in a working methanol to hydrocarbon catalyst. The INS measurements exploit the sensitivity of the technique to hydrogenous species. In comparison with infrared spectroscopy, the technique suffers from lower spectroscopic resolution, but offers several advantages in principle. Analysis can be undertaken directly (as here) on catalyst samples extracted from a working reactor. In particular, there is no restriction on catalyst particle size, or any need to undertake specific sample preparation measures. Furthermore, there is no restriction on the extent of coke formation that can be tolerated before spectra become obscured, and the full spectroscopic range of vibrational frequencies is accessible without obscuration from zeolite framework modes. The catalyst sample analysed here was obtained at a relatively early stage in the reaction profile, but a hydrocarbon pool is clearly already present. We will report later on similar analyses carried out on catalysts reacted for longer periods of time-on-stream, where the advantages over infrared spectroscopy should be even more evident. INS has the potential to be able to correlate catalytic performance with the degree and form of retained surface species. Such information will be valuable in extending our understanding of a role for a hydrocarbon pool in MTG and MTO chemistry.
